# Dynamic stability of gut microbiota in elite volleyball athletes: microbial adaptations during training, competition and recovery

**DOI:** 10.3389/fspor.2025.1662964

**Published:** 2025-09-03

**Authors:** Junior Carlone, Saverio Giampaoli, Eugenio Alladio, Gioele Rosellini, Filippo Barni, Elena Salata, Attilio Parisi, Alessio Fasano, Antonio Tessitore

**Affiliations:** ^1^ Department of Neurosciences, Biomedicine and Movement, University of Verona, Verona, Italy; ^2^ Department of Movement, Human and Health Sciences, University of Rome “Foro Italico”, Rome, Italy; ^3^ Division of Pediatric Gastroenterology and Nutrition, Department of Pediatrics, Massachusetts General Hospital for Children and Harvard Medical School, Boston, MA, United States; ^4^ Department of Chemistry, University of Turin, Turin, Italy; ^5^ Biology Section, RIS Rome, Italy; ^6^ Department of Nutrition, Harvard T.H. Chan School of Public Health, Boston, MA, United States; ^7^ European Biomedical Research Institute of Salerno, Salerno, Italy

**Keywords:** gut microbiota, microbial stability, elite athletes, volleyball, training and competition periodization

## Abstract

**Introduction:**

To examine how different weekly training and competition schedules influence gut microbiota composition in elite volleyball players, investigating the relationship between training and competition demands, recovery periods and microbial dynamics to identify potential biomarkers for training load and recovery status assessment.

**Methods:**

Seven elite athletes from the Italian Men's SuperLega Championship (age: 26.5 ± 4.5 years; weight: 96 ± 11 kg; height: 200 ± 0.1 cm; BMI: 24 ± 1.9) were monitored at four timepoints over eight weeks Regular Season periods (T0, T1), Rest Period (T2) and International Tournament Period (T3). Faecal samples underwent 16S rRNA sequencing analysis, with concurrent Mediterranean Diet adherence and Stool Consistency assessments. Repeated measures ANOVA and one-way ANOVA were performed to evaluate microbial abundance changes.

**Results:**

16S rRNA sequencing revealed Firmicutes predominance (41.22–76.03%), followed by Actinobacteria (9.66–54.45%) and Bacteroidetes (0.73–26.56%). The Firmicutes/Bacteroidetes ratio fluctuated in response to training intensity and competition 6:1 during T0 and T1, decreasing to 3:1 during T2 and returning to 5:1 during T3. Dominant bacterial families included Ruminococcaceae (26.97–28.3%), Bifidobacteriaceae (17.46–22.92%) and Lachnospiraceae (9.66–12.61%). Significant enrichment of Rikenellaceae abundance occurred during Rest Periods (*p* < 0.05). *α*-Diversity remained stable despite individual variation. Mediterranean diet adherence declined during Regular Season Periods (T0: 6.3 ± 1.5, T1: 5.5 ± 0.8), while stool consistency gradually improved.

**Discussion:**

Despite overall stability, elite athletes gut microbiota adapted to volleyball varying training demands primarily via Firmicutes/Bacteroidetes ratio modulations and Rikenellaceae enrichment during Recovery Periods. These microbial alterations represent potential biomarkers for assessing training load and recovery status. Additional investigation is necessary to elucidate how these microbial dynamics influence athletic performance outcomes.

## 1 Introduction

In recent years, the gut microbiota has emerged as a key factor influencing not only general health but also, potentially, athletic performance. However, the effects of training periodization and competition in elite volleyball on gut microbial composition and dynamics remain insufficiently explored. Understanding how microbial dynamics influence athletic performance requires an initial exploratory analysis, followed by a rigorous and comprehensive characterization of the functional and taxonomic properties of the gut microbiota. This dynamic ecosystem exhibits unique individual profiles that perform multiple essential functions, including metabolic regulation, barrier integrity maintenance, immune response modulation, pathogen defence, intestinal homeostasis, biosynthesis, nutrient absorption and epithelial integrity preservation (1–6). Additionally, it exerts a significant influence on drug and xenobiotic metabolism (1, 2). Gastrointestinal bacterial colonization occurs during infancy and progressively stabilizes throughout childhood in response to lifestyle factors (7, 8). Although intestinal bacterial diversity demonstrates positive correlations with both general health and enhanced physical performance, the considerable individual variation precludes the establishment of a universal healthy microbiome model (9–11). Sustaining an optimal microbiota-host equilibrium remains fundamental to overall health (12). Adult gut microbiota architecture maintains relative stability while exhibiting significant interpersonal variation, resulting in distinct enterotypes shaped by dietary patterns, physical activity levels, body mass index, lifestyle factors and environmental exposures (8, 10, 13–16). Elderly populations characteristically demonstrate lower *α*-diversity, with age-associated dysbiosis compromising immune function and elevating susceptibility to various pathological conditions (5, 17). Lifestyle factors, sports activities and individual characteristics influence microbiota composition (17).

While growing evidence supports exercise benefits on microbial diversity, the specific impact of intense physiological demands in elite volleyball on gut microbiota composition across different competitive periods remains poorly understood. Elite sports can reshape gut microbiota composition through intense training, probiotic supplementation and pharmacological interventions (7, 18, 19). Excessive training loads may impair muscle adaptation processes due to dysbiosis associated with oxidative stress and chronic inflammation (20), emphasizing the importance of optimizing gut microbiota composition to modulate immunity and reduce oxidative stress. Elite athletes exhibit enhanced microbial diversity and immunological efficiency compared to sedentary populations (18–23). Microbiome profiling provides valuable insights for monitoring athletic performance, health status and energy availability (24). Intestinal dysbiosis can compromise training adaptations, elevate inflammatory biomarkers and reactive oxygen species generation, accelerate macromolecule catabolism through free radical activity and contribute to skeletal muscle atrophy. Strenuous exercise can negatively modify gut microbiota composition, potentially compromising performance outcomes (25). Despite increasing scientific interest in the role of gut microbiota in sports performance (22), consensus regarding the specific effects of training and competition remains elusive. Observed variations may be attributed to differences between aerobic and anaerobic activities, endurance vs. strength sports, periodization and competitive season phase. Team sports characteristically feature distinct Off-season and In-season periods, as exemplified by elite volleyball competition. This investigation addresses the current lack of data concerning gut microbial health in relation to exercise intensity among elite volleyball athletes during competitive season phases.

Therefore, the primary aim of this pilot study was to investigate how different training and competition phases influence gut microbiota composition in elite volleyball athletes during a Competitive Season. Specifically, we aimed to characterize the temporal dynamics of gut microbiota during a portion of the Regular Season, Rest Period and International Friendly Tournament, to identify potential microbial biomarkers associated with training load variations and to examine lifestyle factors of athletes during these periods. This study focused on the taxonomic characterization of gut microbiota, providing a potential preliminary foundation for future investigations that may explore the functional and metabolic aspects of these microbial changes through metagenomic and metabolomic approaches.

We hypothesized that highly competitive periods may alter gut microbiota composition due to increased physiological demands, consistent with Akazawa et al. observations on the influence of training periodization on gut microbial profiles in elite athletes (26).

## 2 Methods

### 2.1 Ethics

This investigation involved human subjects and received approval from the Ethics Committee of the University of Rome “Foro Italico” (CAR 240/2025) in accordance with the Declaration of Helsinki. All participants provided written informed consent prior to study enrollment.

### 2.2 Participants

Seven elite male volleyball players (*n* = 7) from an Italian SuperLega team voluntarily participated in this study. The international cohort included athletes born in France (*n* = 1), Italy (*n* = 4) and Serbia (*n* = 2) (Table 1). All participants provided written informed consent and data collection adhered to both the principles of the Declaration of Helsinki and applicable national privacy regulations. Exclusion criteria were verified, none of the participants reported recent injuries, acute or chronic illnesses, gastrointestinal comorbidities or treatment with antibiotics, prebiotics, probiotics or postbiotics during the six-month period preceding study enrollment.

**Table 1:** Characteristics of participants.

Characteristic	Value (mean ± SD)
Experience (years)	17 ± 5
Age (years)	26.5 ± 4.5
Ethnicity	White (100%)
Environment	Urban (100%)
Weight (kg)	96 ± 11
Height (cm)	200 ± 0.1
Body Mass Index (kg/m^2^)	24 ± 1.9

### 2.3 Design and sample collection

**Figure 1:**
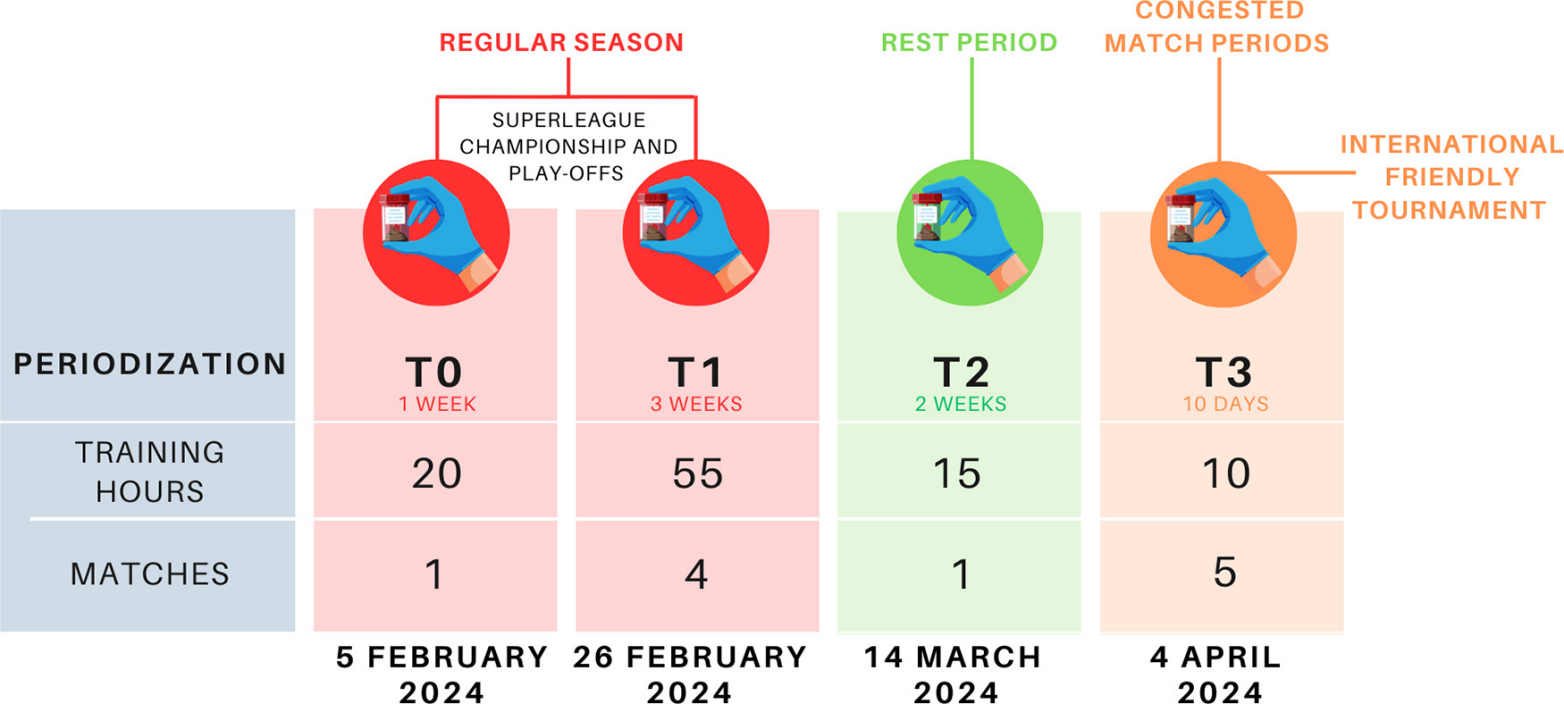
Sampling programme.

This study was conducted during the In-Season competitive Period of the Italian SuperLega Championship. Data collection spanned eight consecutive weeks from February to March 2024, including the 17th to 22nd matchday of the Regular Season and included four sampling timepoints: during the Regular Season (T0, T1), following the Rest Period (T2) and during the International Friendly NAS Sports Tournament 2024 (T3) preceding the play-off competition (Figure 1).

At each time point, fecal samples were collected while participants concurrently completed the Short Mediterranean Diet Questionnaire (SMDQ) through online forms (27). The SMDQ was supplemented with questions about smoking habits, alcohol consumption and Non-Steroidal Anti-Inflammatory Drug (NSAIDs) usage. Participants also recorded stool consistency using the Bristol Stool Form Scale (BSFS) (28).

### 2.4 Nutritional assessment

Participants documented dietary intake at all four assessment intervals utilizing the online SMDQ, from which Mediterranean dietary adherence indices were derived. Concurrently, they evaluated their stool consistency employing the BSFS at each sampling time point. An expert nutritionist provided detailed instructions for accurate questionnaire completion. Temporal changes in stool consistency were subsequently analysed.

### 2.5 NGS analysis of fecal samples

Fecal samples were self-collected by participants using sterile swabs (COPAN, Brescia, Italy) after standardized training. Samples were immediately stored at −20°C and transported to the laboratory under temperature-controlled conditions. Data collection was performed across four timepoints: T0 (February 6, 2024), T1 (February 27, 2024), T2 (March 14, 2024) and T3 (April 5, 2024). Microbiota profiling was performed using 16S rRNA gene amplicon sequencing. DNA extraction followed the protocol established by Giampaoli et al. (29), with mechanical lysis and purification. To ensure elevated reproducibility standards and minimize operator-dependent errors, a fully automated robotized protocol was implemented for DNA extraction, library preparation, chip preparation and sequencing procedures (30). Library preparation was conducted using the Ion Chef™ Instrument (Thermo Fisher Scientific) with Precision ID DL8 Reagents (A32926), Precision ID DL8 Solutions (A30934) and the WG00607 16S Ion AmpliSeq™ custom primer pool focused on eight hypervariable regions, utilizing 22 amplification cycles. Barcoding employed the Precision ID IonCode Adapters (A33419) and libraries were loaded onto Ion 540™ Chips (A27765). Libraries were quantified using the Ion Library TaqMan™ Quantitation Kit on a 7500 RealTime PCR System (Applied Biosystems), with libraries diluted 1:50 and 1:500 for accurate quantification. Target library concentration was set at 50 pM for optimal sequencing performance. Sequencing was carried out on the Ion GeneStudio™ S5 System (Thermo Fisher Scientific), following automated chip preparation on the Ion Chef™. Sequencing runs were performed using the “16S Metagenomics” application on Torrent Suite Software. Data analysis was performed with Ion Reporter Software (AmpliSeq Microbiome Health w1.3 workflow, version 5.20) for taxonomic classification.

*α*-Diversity was calculated using the Shannon index via GAIA software (Sequentia Biotech, version 2.0), with values ranging from 2.72 to 4.15 (3.56 ± 0.48), while *β*-diversity was evaluated using Bray-Curtis dissimilarity metrics and visualized through Principal Coordinates Analysis (PCA) (31, 32). Diversity measures were assessed across four timepoints (T0, T1, T2, T3). The robotized sequencing approach and bioinformatic workflow were designed to ensure reproducibility and reliability in accordance with best practices in microbiome research (33).

### 2.6 Statistics

Although the small sample size limited reliable normality testing, parametric tests were used when assumptions appeared reasonable, with non-parametric alternatives applied when necessary. Temporal effects on microbiota composition were evaluated using repeated measures Analysis of Variance (ANOVA) on longitudinal data collected from 7 subjects across 4 distinct timepoints. This methodological approach accounted for correlations within subject observations while simultaneously examining main time effects and potential subject-time interactions. Mauchly's sphericity test was utilized to assess homogeneity of variance-covariance assumptions; in instances where these assumptions were violated, Greenhouse-Geisser corrections were implemented. Statistical analyses were conducted using Jamovi software (version 2.3.20) for descriptive statistical calculations and ANOVA implementation, with supplementary analyses conducted in RStudio environment (version 2023.09.1 + 522). Significant main effects (*p* < 0.05) were subsequently investigated through Bonferroni-corrected *post-hoc* comparisons to identify specific between-timepoint differences. In addition, repeated measures ANOVA was used to assess temporal changes in the SMDQ and BSFS scores. Greenhouse–Geisser corrections were applied when sphericity was violated. Moreover, one-way ANOVA was applied at the group level to assess differences in specific microbial taxa across the four timepoints (T0–T3). This univariate approach was introduced as a simplified and more interpretable alternative to ANOVA–Simultaneous Component Analysis (ASCA), which did not yield conclusive patterns in this dataset. While this method does not account for intrasubject correlations inherent to repeated measures designs, it allowed for an exploratory evaluation of general temporal trends at the group level. The results from this analysis were interpreted with caution and contextualized alongside the findings from repeated measures ANOVA and PCA. Prior to primary analyses, data preprocessing procedures enhanced dataset quality by applying a 40% filtering threshold to exclude low-prevalence parameters; specifically, variables exhibiting more than 40% zero values were excluded from further analysis. The remaining data were standardized using autoscaling procedures, centering and scaling to unit variance.

## 3 Results

### 3.1 16s rRNA metabarcoding profile

**Figure 2:**
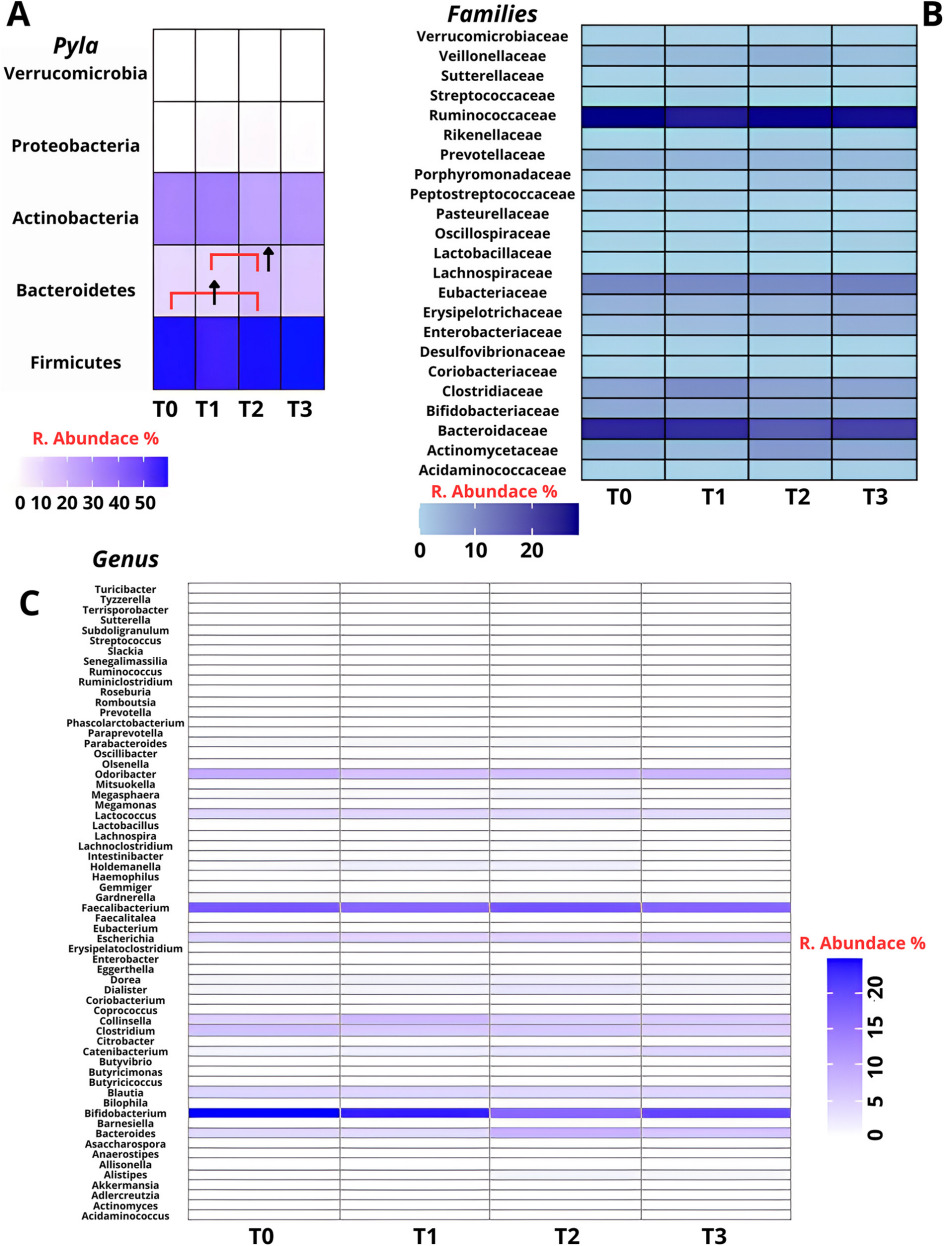
Relative abundance of gut microbiota across four timepoints (T0–T3) at three taxonomic levels: Phyla **(A)**, family **(B)** and genus **(C)**. Firmicutes represented the most prevalent phylum throughout the study, while temporal fluctuations were observed in Bacteroidetes and Actinobacteria. At the family level, Ruminococcaceae and Bifidobacteriaceae were consistently abundant. At the genus level, Bifidobacterium and Faecalibacterium were predominant. Statistical analysis by repeated measures ANOVA revealed no significant differences over time (*p* > 0.05). (↑ = Increase).

Phylum level analysis showed Firmicutes predominance (41.22–76.03%), followed by Actinobacteria (54.45%–9.66%) and Bacteroidetes (26.56%–0.73%). Average composition at T0 consisted of Firmicutes (58.7 ± 10%), Bacteroidetes (9.7 ± 6.7%), Actinobacteria (31 ± 11.4%), Proteobacteria (0.4 ± 0.5%) and Verrucomicrobia (0.2 ± 0.4). At T1 showed Firmicutes (56.3 ± 10.3%), Bacteroidetes (9.5 ± 5.9%), Actinobacteria (32 ± 11.6%), Proteobacteria (2.0 ± 2.5%) and Verrucomicrobia (0.1 ± 0.2). At T2 Firmicutes (57.7 ± 14.8%), Bacteroidetes (17 ± 7.1%), Actinobacteria (23.6 ± 9.6%), Proteobacteria (1.6 ± 1.5%) and Verrucomicrobia (0.1 ± 0.3) and at T3 Firmicutes (60.5 ± 10.1%), Bacteroidetes (12.8 ± 6.7%), Actinobacteria (25.8 ± 14.8%), Proteobacteria (0.8 ± 1%) and Verrucomicrobia (0 ± 0) (Figure 2A). Although an increase in Bacteroidetes abundance was observed between both T0–T2 and T1–T2 time intervals, ANOVA revealed these elevations did not reach statistical significance (*p* = 0.15) (Figure 2A). The Firmicutes/Bacteroidetes (F/B) ratio fluctuated with training demands: 6:1 during high intensity Competitive Periods (T0: 58.7/9.7; T1: 56.3/9.5), 3:1 during Rest Period (T2: 57.7/17) and 5:1 during Tournament Play Period (T3: 60.5/12.8), while Firmicutes/Actinobacteria (F/A) ratio remained stable at 2:1. Family level analysis revealed Ruminococcaceae predominance (T0:28.3 ± 7.4, T1:26.97 ± 13.45, T2:28.3 ± 11.09, T3:28.14 ± 7.14), followed by Bifidobacteriaceae (T0:22.92 ± 12.55, T1:21.42 ± 15.67, T2:17.46 ± 12.04, T3:19.36 ± 16.50) and Lachnospiraceae (T0:10.44 ± 3.18, T1:10.27 ± 3.02, T2:9.66 ± 3.28, T3:12.61 ± 3.10) (Figure 2B). At genus level *Bifidobacterium* was most abundant (T0:24.83 ± 12.81, T1:21.38 ± 15.72, T2:16.55 ± 11.07, T3:19.36 ± 16.50), with *Faecalibacterium* (T0:17.76 ± 6.39, T1:17.62 ± 10.45, T2:18.46 ± 8.19, T3:17.72 ± 6.00) and *Ruminococcus* (T0:8.47 ± 2.90, T1:6.57 ± 3.22, T2:6.69 ± 3.21, T3:7.91 ± 2.70) (Figure 2C).

**Figure 3:**
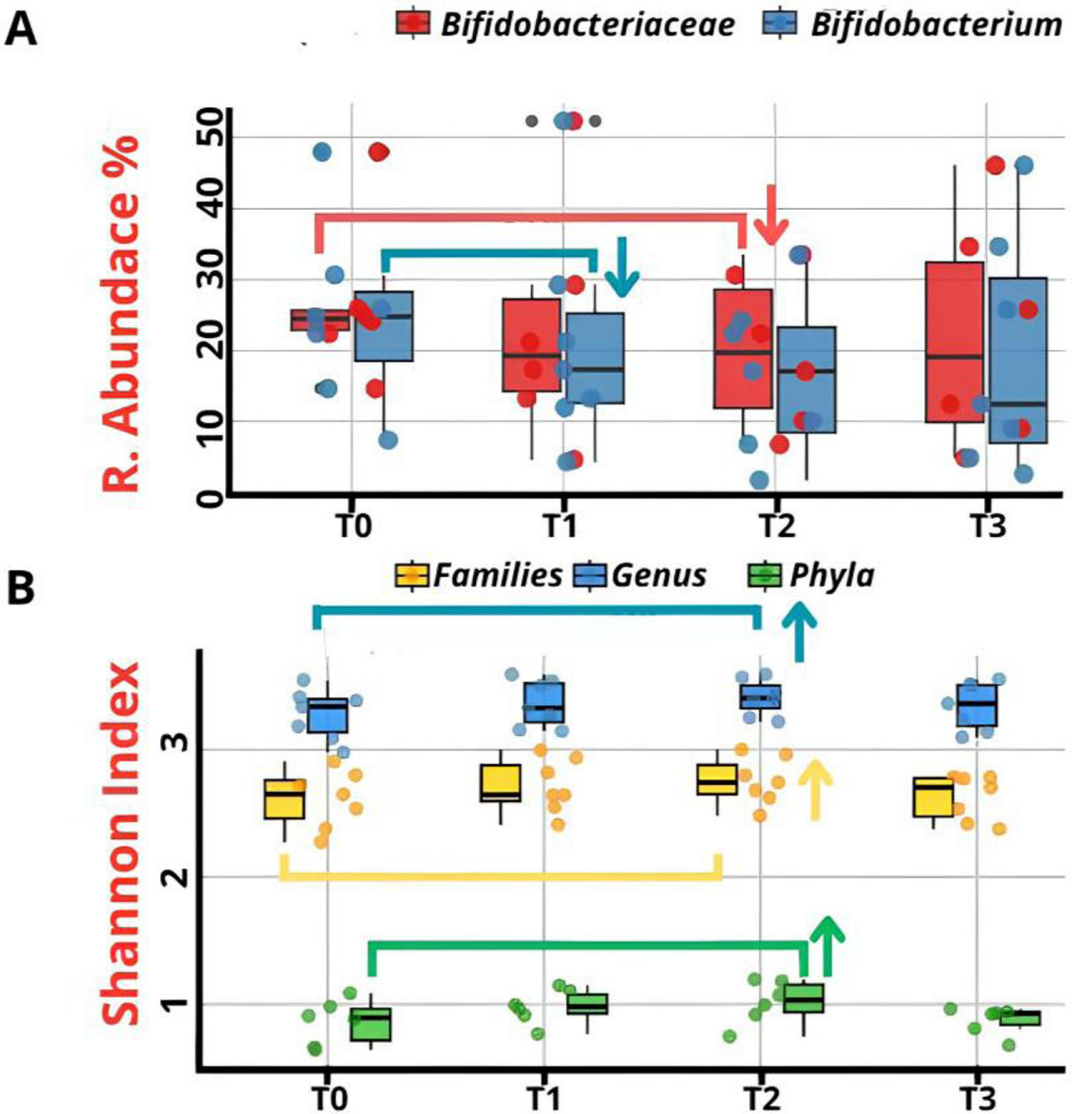
**(A)** Comparison of the relative abundance of Bifidobacteriaceae at family level and Bifidobacterium at genus level across four sampling timepoints (T0–T3). **(B)** Comparison of *α*-diversity values (Shannon index) at the Phylum, Family, and Genus levels across all time points (T0–T3). No statistically significant differences were observed (*p* > 0.05). (↑ = Increase; ↓ = Decrease).

Despite temporal variations, Bifidobacteriaceae showed no statistically significant differences (*p* = 0.08) by repeated measures ANOVA (Figure 3A). Similarly, *Bifidobacterium* exhibited no significant differences (*p* = 0.06) according to the Kruskal–Wallis testing, despite its prominent expression (Figure 3A). *α*-Diversity, assessed using Shannon index, remained stable across timepoints at Phylum level (T0:0.86 ± 0.16, T1:0.96 ± 0.13, T2:0.99 ± 0.17, T3:0.87 ± 0.10; *p* = 0.09), Family level (T0:2.60 ± 0.22, T1:2.71 ± 0.21, T2:2.75 ± 0.18, T3:2.62 ± 0.17; *p* = 0.26) and Genus level (T0:3.27 ± 0.19, T1:3.36 ± 0.18, T2:3.40 ± 0.14, T3:3.34 ± 0.18; *p* = 0.29) (Figure 3B). Though not temporally significant, marked interindividual diversity variation was observed.

**Figure 4:**
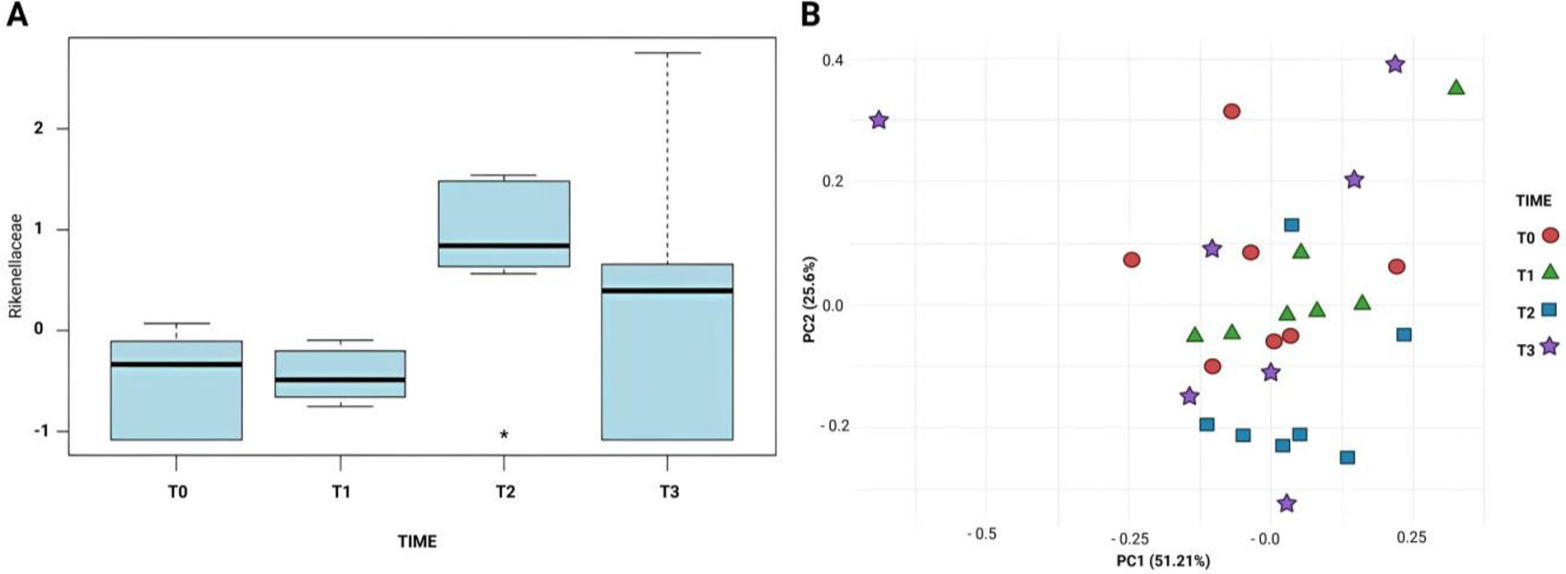
**(A)** Comparison of the relative abundance of Rikenellaceae across timepoints (T0–T3), analysed by one-way ANOVA. A statistically significant increase was observed at T2 (*p* < 0.05). **(B)** Principal Coordinates Analysis (PCA) plot based on normalized data, showing moderate temporal separation in microbial community composition.

One-way ANOVA was conducted at both family and genus taxonomic levels to identify significant differences in microbial composition. Data underwent two-step normalization, removing variables with >40% zero values, then auto scaling the remaining data by centering around means and scaling by standard deviations. This transformation removed scale differences and ensured equal feature contribution to the statistical analysis. The primary objective was to evaluate time-associated microbial variations associated with sampling timepoints. At Family level, ANOVA revealed statistically significant Rikenellaceae abundance increase at T2 (*p* < 0.05), confirmed by *post-hoc* analysis that showed significant pairwise differences, establishing Rikenellaceae as a key contributor to temporal microbial variation (Figure 4A).

PCA on normalized data revealed moderate separation between timepoints in microbial compositions. Though not strongly pronounced, principal component space inspection showed T2 microbial profiles diverging from earlier stages, suggesting underlying community structure shifts. This moderate separation supports ANOVA findings by highlighting temporal microbial dynamics (Figure 4B), underscoring Rikenellaceae significance as a temporally influenced taxonomic group.

### 3.2 Dietary and intestinal health assessment

Mediterranean diet adherence, measured through the SMDQ, showed a non-significant negative fluctuation during T1 (T0: 6.3 ± 1.5; T1: 5.5 ± 0.8; T2: 5.6 ± 1.1; T3: 5.5 ± 1.1). Repeated measures ANOVA revealed no significant effect of time (*p* = 0.23). All athletes maintained a minimum of 3/5 daily meals throughout the investigation Period. With SMDQ scale ranging from 0 to 9, participants displayed intermediate adherence values (Table 2). Stool consistency, measured using the BSFS scale, showed a non-significant gradual increase over time (T0: 2.9 ± 1.1; T1: 3.0 ± 0.8; T2: 3.3 ± 0.8; T3: 3.3 ± 0.8), without a statistically significant time effect (*p* = 0.33), as determined by repeated-measures ANOVA. Although the assumption of sphericity was violated (Mauchly's test *p* = 0.013), Greenhouse–Geisser correction confirmed the non significance of the result (*p* = 0.32).

**Table 2:** Participants’ life habits.

Variables	Value (mean ± SD)	Value (mean ± SD)	Value (mean ± SD)	Value (mean ± SD)
Time	T0	T1	T2	T3
SMDQ	6.3 ± 1.5	5.5 ± 0.8	5.6 ± 1.1	5.5 ± 1.1
BSFS	2.9 ± 1.1	3.0 ± 0.8	3.3 ± 0.8	3.3 ± 0.8
Cigarette Smoke	4 of 7 (57.1%)	4 of 7 (57.1%)	4 of 7 (57.1%)	4 of 7 (57.1%)
Weekly Alcohol	3 of 7 (42.9%)	3 of 7 (42.9%)	4 of 7 (57.1%)	4 of 7 (57.1%)
Weekly NSAIDs	3 of 7 (42.9%)	2 of 7 (28.6)	1 of 7 (14.4%)	0 of 7 (0.0%)

SD, standard deviation; SMDQ, short mediterranean diet questionnaire; BSFS, bristol stool form scale; NSAIDs, non-steroidal anti-inflammatory drugs*.*

Analysis of lifestyle factors revealed consistent regular cigarette smoking (57.1%) across all timepoints, while alcohol consumption (>1 drink/week) increased during Rest and Tournament Periods (57.1%). NSAIDs usage decreased progressively from regular season (T0: 57%, T1: 29%) through Rest Period (T2: 14%) to tournament phase (T3: 0%) (Table 2).

## 4 Discussion

### 4.1 Main results

**Figure 5:**
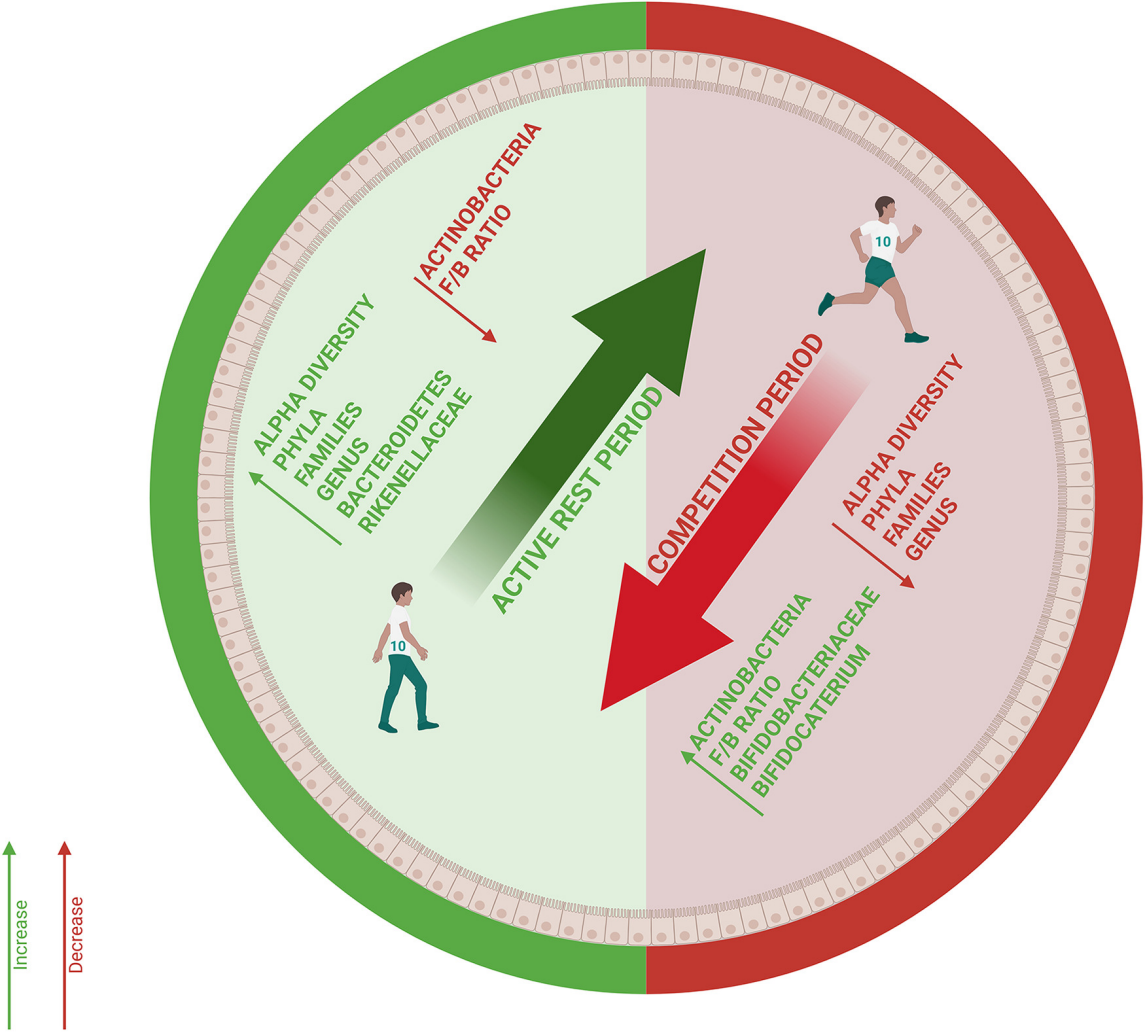
Intestinal microbiota dynamics in elite volleyball athletes. The diagram illustrates variations in microbial composition between active Rest Period (green) and Competitive Periods (red). (F/B = Firmicutes/Bacteroidetes ratio).

This pilot study reveals three potential patterns in gut microbiota dynamics of elite volleyball athletes, including fluctuations in the F/B ratio that may reflect the energetic demands of different competitive phases, significant enrichment of Rikenellaceae during recovery (*p* < 0.05) and general microbiota stability suggesting that elite athletes may possess resilience of the microbial ecosystem optimized for elite athletic performance. The effects of intense physical performance on gut microbiota composition remain incompletely characterized to date and to our knowledge, gut microbiota dynamics during part of a Competitive Season among elite volleyball athletes have not been previously investigated (34). Our results partially align with the literature, showing Firmicutes predominance with prevalent Ruminococcaceae and Lachnospiraceae and elevated populations of *Faecalibacterium* and *Ruminococcus* (35, 36) (Figure 5).

### 4.2 Physiological interpretation

We observed elevated levels of Actinobacteria, particularly Bifidobacteriaceae, which merit attention given their potential role in immunomodulation, gut-brain axis signalling, metabolic regulation and barrier function maintenance, including possible modulation of intestinal permeability in response to physiological stressors (37, 38). The mechanistic significance of these changes emerges when considering that increased intestinal permeability is partially supported by the observed trend, where *Collinsella* increased during high-intensity raining and Competition Periods, T0 and T1 (35). This connection could indicate, albeit preliminarily, how acute physical demands can modulate intestinal barrier integrity through specific microbial taxa, thus likely influencing systemic homeostasis (39).

We observed an increase in Bacteroidetes during the Rest Period, which could be associated with their potential protective anti-inflammatory role through which they limit lipopolysaccharide (LPS) production (40). This supposition could represent an adaptive mechanism through which the gut microbiota may actively support recovery, shifting from an energy production oriented configuration, dominated by Firmicutes, to an anti-inflammatory configuration optimized for possible tissue repair. Furthermore, although still understudied, we observed a significant increase in Rikenellaceae during the Rest Period, which aligns with what Hughes et al. described, where they reported a reduction of this family in murine models exposed to prolonged high levels of physical activity (9). This may suggest that, since Rikenellaceae are also associated with *Butyrococcus*, they could exert a protective effect in this context (41). The physiological relevance of this enrichment could become evident considering the metabolic capacity of these taxa, where although they are minor producers compared to other genera, they can produce succinate among other organic acids, an important metabolite in mitochondrial function regulation and cellular energy metabolism (42, 43). The functional significance of succinate in the context of athletic recovery could also be supported by its multiple properties. Succinate has been defined as an epigenetic modulator, oncometabolite, hypoxic response mediator, post-translational mechanism mediator, reactive oxygen species metabolism regulator, endocrine and paracrine modulator and inflammation signalling molecule (44). As a signalling molecule, succinate can exert different actions depending on the context, both pathological and physiological, directly explaining why the relative enrichment of succinate-producing taxa during recovery phases suggests a possible adaptive role of the microbiota in supporting the organism's energetic and regenerative response. However, further metabolomic studies are necessary to validate the functional significance of succinate production by Rikenellaceae in athletic recovery contexts.

Supèporting the overall pattern of microbial adaptation to athletic recovery, Porphyromonadaceae also demonstrated increased abundance during the Rest Period, consistent with their association with cardiac function and negative correlation with metabolic disorders (45). Conversely, the F/B ratio decreased during the Rest Period and increased during Periods where intensity was higher, T0, T1 and T3. The F/A ratio remained stable, likely reflecting the critical role of Actinobacteria in energy metabolism processes. *α*-Diversity increased non significantly during the Rest Period, while decreasing during intensive Training and Competition Periods, as it appears responsive to external environmental stimuli (46). Additionally, the coordinated production of beneficial metabolites is evidenced by the fact that, although short-chain fatty acids (SCFA's) were not directly measured, the populations found to be most elevated including Bifidobacterium, *Faecalibacterium, Ruminococcus,* and Bifidobacteriaceae in athletes suggest enhanced production of acetate, butyrate, propionate and succinate, thus likely supporting microbiota homeostasis across these various phases (47, 48). These physiological mechanisms of microbial adaptation could have implications for monitoring and management of elite athletes.

### 4.3 Practical implications

Translating these mechanisms into practical applications for athletes, the primary advantage of the study derives from examining elite volleyball athletes during a critical phase of the Competitive Season, providing possible insights into gut microbiota dynamics under competitive conditions. Overall, we observed microbial community stability, presumably optimized for maintaining physiological homeostasis, while simultaneously identifying key compositional fluctuations corresponding to training periodization and programming cycles in elite volleyball championship. These consistent patterns establish a possible direct connection between microbial observations and potential applications in athletic monitoring between gut microbiota and skeletal muscle during variable competitive demands, with specific biomarkers emerging as possible potential indicators for monitoring training load and recovery status of elite volleyball athletes (49, 50).

### 4.4 Strengths and limitations

The primary strength of the study derives from examining elite volleyball athletes during a critical phase of the Competitive Season, providing the first evidence on gut microbiota dynamics under real competitive conditions in this sport (51). Additionally, the within-subject longitudinal design used in this study represents a robust methodological approach that reduces interindividual variability and improves interpretability of microbiota changes across training and competition phases (52). This approach allowed identification of specific microbial patterns, such as F/B ratio fluctuations and Rikenellaceae enrichment, which might otherwise be masked by between-subject variability.

However, several methodological limitations influence interpretation of the results. The limited sample size restricts statistical power to detect significant differences in less abundant taxa and may explain why some observed patterns such as for Bifidobacteriaceae did not reach statistical significance despite biologically relevant trends. Furthermore, exclusive use of 16S rRNA sequencing prevents direct functional characterization of metabolic pathways, limiting for example conclusions about proposed mechanisms for succinate production by Rikenellaceae during recovery. The absence of inflammatory biomarkers and physical performance metrics in the study design also prevents the possibility of establishing direct correlations between observed microbial changes and physiological or performance outcomes. The presence of confounding factors in the cohort represents an additional interpretative limitation. Regular cigarette smoking in 57.1% of athletes can specifically influence gastrointestinal health and microbiota composition, affecting immune function and specific training effects, as well as increased alcohol consumption (>1 drink/week) during Rest and Tournament Periods (42.9%–57.1%) could also contribute to causing these negative interferences for human health and performance (53, 54). Furthermore, NSAID's usage showed progressive reduction, ceasing completely during the International Friendly Competition, highlighting the need to increase awareness among athletes and support staff about chronic effects of medications on gut microbiota composition (55). Due to the limited sample size and high prevalence of smokers, it was not possible to perform statistical analyses to evaluate the direct impact of these factors on gut microbiota composition. Future investigations with larger cohorts should include these analyses to explore in greater depth the potential associations between lifestyle factors and actual metabolite production, allowing more comprehensive understanding of mechanistic relationships between microbial composition and athletic performance (56).

Despite these limitations, the coherence of patterns observed across participants and the longitudinal design of the study suggest that training and competition effects are sufficiently robust to emerge despite confounding factors. Furthermore, the prevalence of smoking and alcohol consumption in our cohort, while representing confounding factors, reflects realistic patterns observed in elite athletes, providing preliminary insights into microbiota adaptations under real rather than ideal conditions.

## 5 Future perspectives

Despite the identified limitations, the consistent patterns observed in F/B ratio and Rikenellaceae enrichment establish a possible preliminary basis for understanding microbial adaptations in elite volleyball athletes. Future directions derive directly from proposed mechanisms and identified methodological limitations, with four priority areas of investigation that could transform elite athlete monitoring. Validation studies with larger cohorts are necessary to confirm the robustness of F/B fluctuations during different Competitive Phases and to characterize the specificity of Rikenellaceae enrichment during the Recovery Phase. These studies should stratify for confounding factors such as smoking, alcohol and NSAID's use to isolate specific training effects and include control groups of athletes from other team sports and sedentary population to distinguish sport specific adaptations from general exercise ones. Additionally, recruitment of elite athletes with completely controlled lifestyles, although very challenging, would allow evaluation of the extent of training effects in the absence of significant confounding factors. Integration of shotgun metagenomics and metabolomic analyses is essential, primarily to validate the hypothesis of succinate production by Rikenellaceae during recovery and to elucidate functional pathways underlying observed adaptations. Specifically, shotgun metagenomics would allow identification of specific genes and metabolic pathways present in these taxa, while metabolomics could directly quantify succinate, butyrate, acetate and propionate in plasma and feces during different training phases. Furthermore, biomarkers of inflammation, intestinal permeability and oxidative stress should be measured to connect microbial changes to physiological outcomes and establish causal relationships between microbiota and athletic performance. Development of microbiota monitoring protocols based on identified biomarkers could provide innovative practical tools for elite sports medicine. The F/B ratio could serve as an indicator of training load and metabolic energetic status for medical staff and coaches, while Rikenellaceae abundance could predict recovery effectiveness and early identify overtraining states during programmed Rest Periods. Future studies should also integrate longitudinal performance assessments with microbiota profiling to better evaluate its predictive and practical utility in athlete monitoring and validate these potential biomarkers against established physiological markers such as heart rate variability (HRV) and cortisol. Finally, randomized controlled trials should explore targeted nutritional interventions to optimize microbial adaptations during different periodization phases. Specific probiotic strategies could be proposed, such as with succinate producing strains, prebiotic interventions to support Rikenellaceae enrichment during recovery and periodized nutritional protocols could be tested for their impact on microbiota composition, recovery biomarkers and athletic performance.

Integration of these approaches could guide development of personalized periodization strategies toward precision sports medicine based on gut microbiota. This would represent a paradigmatic shift in elite athlete management, where microbiota profiling could be implemented as a complementary physiological monitoring tool to optimize performance, prevent overtraining states and accelerate recovery through targeted interventions. The goal is to establish gut microbiota as a sensitive indicator for identifying incipient overtraining states in elite athletes, opening new frontiers in modern sports medicine.

## 6 Conclusion

Integrating results, mechanisms, and practical considerations, this pilot investigation provides innovative insights into the relationship between gut microbiota composition and elite volleyball performance, although further studies are needed to confirm these preliminary findings. Our results demonstrate that intestinal microbial communities can respond to different training and competition periods with remarkable homeostatic stability while simultaneously exhibiting significant fluctuations in F/B ratio and Rikenellaceae abundance. The coherence of these patterns across the cohort establishes the basis for practical applications, where Rikenellaceae members appear to be interconnected with other bacteria such as Butyrococcus, which have been associated with physical performance optimization, suggesting potential synergistic roles within the gut microbial ecosystem (41, 57). In summary, these preliminary results establish a possible scientific foundation for understanding how the gut microbiota of elite volleyball athletes can dynamically adapt to competitive demands, providing new possible perspectives for innovative practical applications in sports medicine.

## Data Availability

The data are not publicly available due to privacy concerns related to human subject’s research and the small sample size of elite athletes that could potentially allow identification. Requests to access the data will be considered by the authors within the constraints of privacy and consent. Requests to access the data can be directed to the corresponding author.
