# Supplementary material for: Dynamic stability of gut microbiota in elite volleyball athletes: microbial adaptations during training, competition and recovery
Source: Front Sports Act Living. 2025 Sep 3;7:1662964. doi: 10.3389/fspor.2025.1662964 (PMC12440923; doi:10.3389/fspor.2025.1662964)
Supplement: Supplementary file 1 [file Datasheet1.pdf]

UNIVERSITÀ di VERONA

UNIVERSITÀ DEGLI STUDI DI ROMA "FORO ITALICO"

Instructions for stool collection

The following instructions should be followed to collect the stool sample:

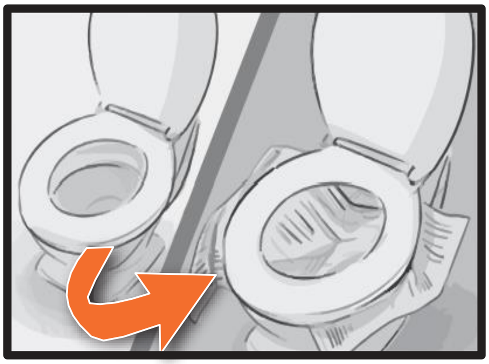

1. Using the provided gloves, place the sterile cardboard Disposable Bedpan in the toilet/bidet in such a way that it retains feces for the time necessary to perform the collection.
2. Unscrew and remove the plug gauge from the container, taking care not to touch anything.

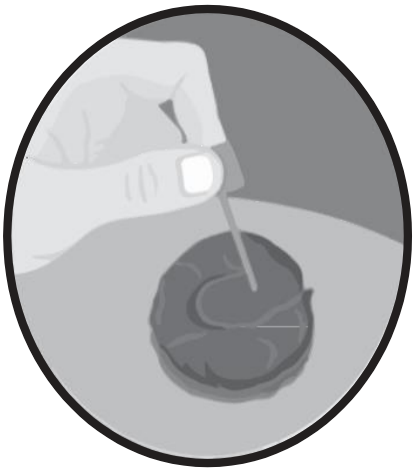

3. Gently rub and soak the upper portion of the faeces using the swab.
4. Close the swab in the plastic casing.
5. **Check that the container bears your identification code for the questionnaire.**
6. Fill in the Form

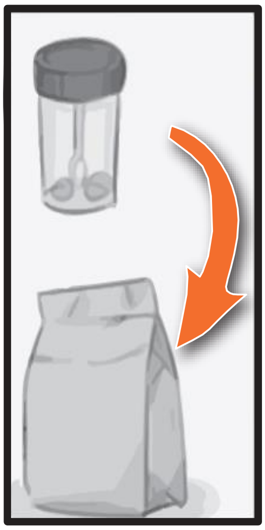

7. Place the container in the marked paper bag.
8. Bring the sealed paper envelope to the same morning's training session.

Notes:

- No special diet is necessary before the test.
- Avoid contaminating faeces with urine.
- Do not use the swab if it is broken or if it has been in contact with objects other than faeces.
